# Supplementary material for: Asymmetric Synthesis of Quaternary Hydantoins via a Palladium-Catalyzed Aza-Heck Cyclization
Source: J Am Chem Soc. 2025 Nov 14;147(49):44692–8. doi: 10.1021/jacs.5c16022 (PMC12703750; doi:10.1021/jacs.5c16022)
Supplement: Supplementary file 2 [file ja5c16022_si_002.zip › All NMR FID Files/1-S-trans/1-S-trans_ProtonNMR/1/pdata/1/email_HO01-166A-1H_1_1.pdf]

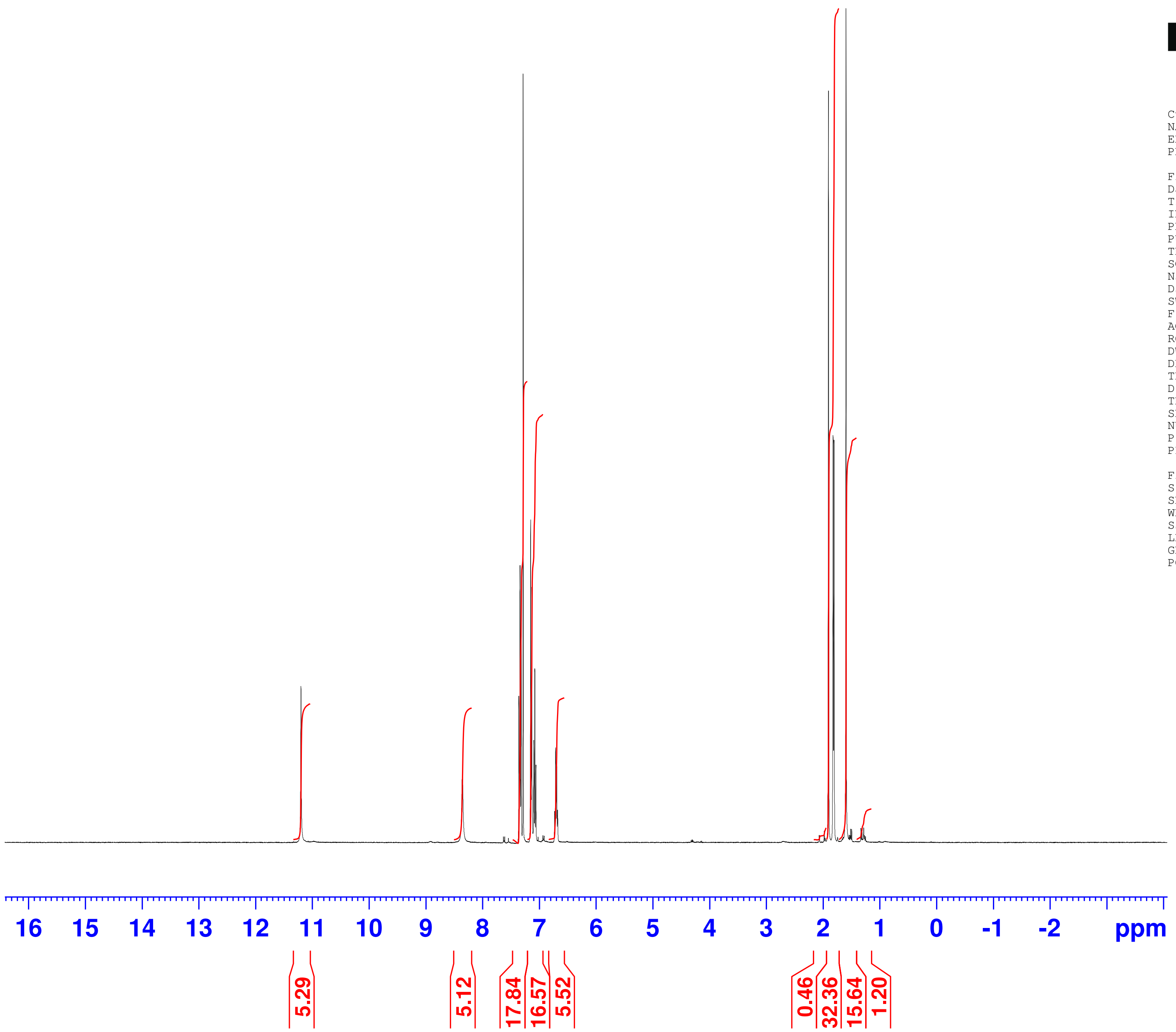

Current Data Parameters
NAME HO01-166A-1H
EXPNO 1
PROCNO 1

F2 - Acquisition Parameters
Date_ 20250628
Time 17.48 h
INSTRUM Avance Neo 400 U of DE Brown Lab
PROBHD Z104450_0412 (
PULPROG zg
TD 65536
SOLVENT CDCl3
NS 16
DS 2
SWH 8196.721 Hz
FIDRES 0.250144 Hz
AQ 3.9976959 sec
RG 101
DW 61.000 usec
DE 13.33 usec
TE 300.0 K
D1 10.00000000 sec
TD0 1
SFO1 400.1324708 MHz
NUC1 1H
P1 15.00 usec
PLW1 8.31639957 W

F2 - Processing parameters
SI 65536
SF 400.1300000 MHz
WDW EM
SSB 0
LB 0.30 Hz
GB 0
PC 1.00
